# Fungal burn wound infection caused by *Fusarium dimerum*: A case series on a rare etiology

**DOI:** 10.1016/j.amsu.2021.102848

**Published:** 2021-09-09

**Authors:** Subaina Naeem Khalid, Nousheen Rizwan, Zeest Ali Khan, Ali Najam, Amin Moazzam Khan, Talal Almas, Tarek Khedro, Vikneswaran Raj Nagarajan, Abdulaziz Alshamlan, Amin Gronfula, Rahaf Alshehri

**Affiliations:** a Shifa College of Medicine, Shifa Tameer-e-Millat University, Islamabad, Pakistan; b Quaid-e-Azam International Hospital, Islamabad, Pakistan; c Royal College of Surgeons in Ireland, Dublin, Ireland

**Keywords:** Fusarium dimerum, Burns, Mold, Fungal wound infection, Case series

## Abstract

**Introduction:**

Fusarium dimerum is a filamentous mold associated with poor outcomes in immunocompromised hosts and burn victims. It can be acquired via inhalation or through skin dehiscence.

**Methods:**

Our work presents a Case series of 8 patients from ages 3–57 years who were admitted with multiple burn wounds over the past 6 months. After initial stabilization measures, they all underwent debridement for the lesions after negative initial fungal cultures. The 44-year-old male was the first patient to develop punched-out eruptions on burn areas 7 days after admission; all the other patients experienced similar lesions during the next 6 days. Tissue cultures of the lesions exhibited Fusarium dimerum growth. The patients were managed accordingly with amphotericin B or voriconazoles. All the patients recovered except the 11-year-old boy, who expired on day 9 due to ARDS and sepsis complications.

**Outcomes:**

Infection with Fusarium dimerum carries a high risk of dissemination in burn infections. Hence, appropriate screening should be carried out via histologic and mycologic diagnostics early in the disease course.

**Conclusion:**

Considering the sparse literature that is available regarding Fusarium infection in burn victims, this study aims to improve the knowledge surrounding different facets of this disease including its epidemiology, diagnosis, management, and the need to maintain high suspicion of this fungal disease in burn patients.

## Highlights

• Fusarium dimerum is a filamentous mold associated with poor outcomes in immunocompromised hosts and burn victims. It can be acquired via inhalation or through skin dehiscence.
• Our work presents a Case series of 8 patients from ages 3–57 years who were admitted with multiple burn wounds over the past 6 months. After initial stabilization measures, they all underwent debridement for the lesions after negative initial fungal cultures.
• Infection with Fusarium dimerum carries a high risk of dissemination in burn infections. Hence, appropriate screening should be carried out via histologic and mycologic diagnostics early in the disease course.

## 1 Introduction

Fusarium dimerum is known to be a non-dermatophytic, filamentous mold [1,2]. It is an exceedingly prevalent cause of mycotic infections, particularly in immunocompromised patients, and is associated with poor outcomes [[3], [4], [5]]. A high risk of dissemination also exists in this population, with the majority being burn victims, patients with hematologic malignancies, and those undergoing chemotherapeutic treatments [3]. Individuals typically acquire infection through the respiratory tract via conidia inhalation or through skin dehiscence following trauma or, more commonly, burns [5]. (see Table 1)

**Table 1:** Case summaries.

Case number	Patient Age (years)	Gender	Burn Area	Initial Treatment*	Initial Cultures	Debridement	Initial admission: ICU/Burn Unit
1	44	Male	20% Head, neck, bilateral forearms and hands	Supportive management with injection Moxifloxacin 400 mg	*Pseudomonas aeruginosa* Acinetobacter baumannii	Multiple rounds done	ICU
2	5	Male	35% Head and neck, Anterior chest, part of lower limbs	Supportive management with meropenem 330 mg I/V 8 hourly	Blood and tissue cultures negative	Alternate day debridement	ICU
3	2.5	Male	15% scalds due to boiling water face and chest and shoulders	Supportive management with injection amoxiclav 300 mg I/V 8 hourly, Injection Amikacin 150 mg I/V 12 hourly	Blood and tissue cultures negative	Done once	Burn Unit
4	11	Male	55% burns with head and neck and trunk and bilateral upper limbs with buttocks and part of thighs	Supportive management with tracheostomy and injection meropenem 500 mg I/V 8 hourly, moxifloxacin 200 mg I/V OD with 1.6 mg I/V 8 hourly	Tissue culture yielded *E. coli* and blood cultures were negative	Excessive debridement and curettage done	ICU
5	57	Female	30% flame face and both and upper and lower limbs	Supportive management with meropenem and moxifloxacin coverage	Blood and Tissue Culture cultures negative	Alternate day debridement	ICU
6	3	Female	22% burns involving trunk, thighs and perineum	Supportive treatment with injection amoxiclav 300 mg I/V 8 hourly	Blood and tissue cultures negative	Alternate day debridement	ICU
7	5	Female	30% flame burns involving upper limbs, abdomen, chest and bilateral lower limbs	Supportive management with meropenem 330 mg I/V 8 hourly	Blood and tissue cultures negative	Alternate day debridement	ICU
8	47	Male	10% deep dermal burns involving hands and both forearms	Supportive management with moxifloxacin I/V OD 400 mg and amoxiclav I/V 1.2 mg 8 hourly	Blood and tissue cultures negative	Done once	Burn Unit

Several species within the Fusarium genus can be identified based on morphology and this often require multi-locus sequence typing (MLST) for confirmation [6,7]. The most encountered Fusarium mold includes the Fusarium solani species complex (FSSC), whereas Fusarium dimerum (FDSC) is a relatively rare plant pathogen [8].

The prevalence of Fusarium infections is suspected to be higher in tropical areas as compared to the rest of the world [5,9,10]. Here, we present a Case series of burn victims who were diagnosed with Fusarium dimerum infections in the past 6 months. The patients discussed are between the ages of 3 and 45 years and had no comorbidities. Case 1 was the first patient that developed lesions, 7 days into hospital admission. Within 6 days of the event, Cases 2 to 8 acquired similar lesions on affected burn areas. Fungal and bacterial cultures along with biopsies were sent for examination for each patient. A Microbiologist on return of four positive fungal cultures and histopathological examination confirmed the results. All these patients were admitted in the Burn unit at the time of development of lesions. To our knowledge, there is currently no literature regarding Fusarium dimerum infection associated with burn victims in Pakistan.

### 1.1 Case 1

**Fig. 1:**
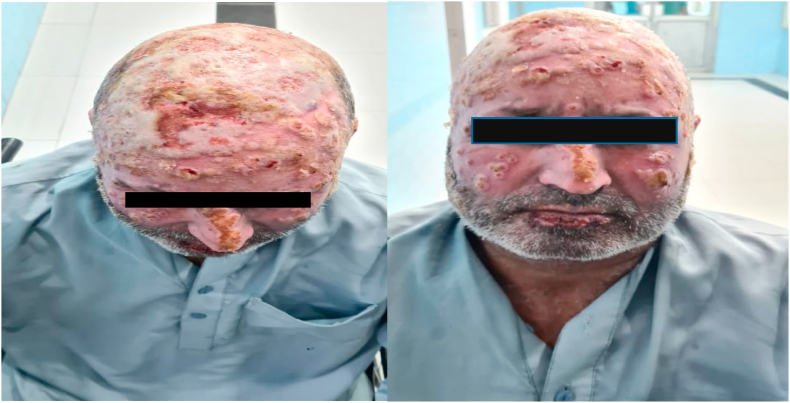
Case 1: 44-year-old male with *Fusarium dimerum* infection.

**Fig. 2:**
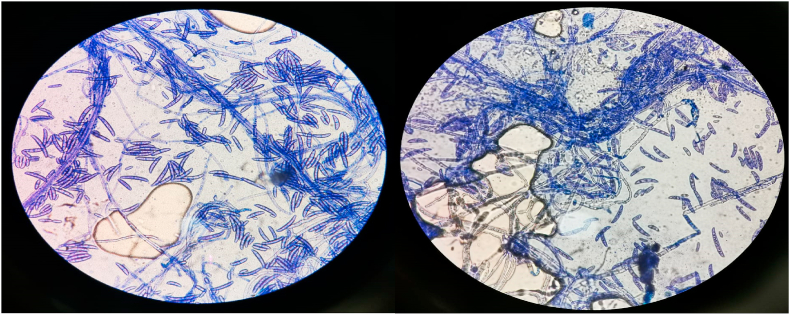
Crescent shaped macroconidia revealed on microscopic examination.

A 44-year-old male with no known comorbidities was admitted to the burn ward with 20% total body surface area (TBSA) flame burns covering his head, neck, bilateral forearms, and hands. The affected area consisted of both deep and partial thickness wounds. He was administered intravenous (IV) normal saline and initial cultures revealed multidrug-resistant *Pseudomonas aeruginosa* and Acinetobacter baumannii sensitive to colomycin. No fungal growths were seen. The patient was started on polymyxin E (Colistin) 2 million IU IV Q8H and meropenem 1 g IV Q8H. Daily cleaning and multiple-wound debridement were performed over a week. Seven days later, the patient started developing high-grade fever and painful, erythematous, punched-out eruptions on superficial and deep burn areas with sparing of intact normal skin tissue (Fig. 1). Initially, it was suspected to be a Case of herpes virus infection. Hence, an appropriate IV acyclovir treatment regime was started. However, the patient did not respond to this treatment, resulting in persistent high-grade fever along with a progressively increasing number of skin lesions. Fungal and bacterial cultures along with biopsies were sent for histopathological examination. The bacterial cultures showed no bacterial growth, however, fungal culture results confirmed the presence of Fusarium dimerum. A total of four differential samples for fungal culture were sent per patient and all samples showed fungal growth. Microscopic analysis of the culture revealed crescent shaped macroconidia, typical for identification of Fusarium (Fig. 2). Histopathological examination carried out by a microbiologist further confirmed presence of mold in tissue samples. After confirmation of the diagnosis, the patient was promptly given a 7-day course of amphotericin B and voriconazole.

### 1.2 Case 2

**Fig. 3:**
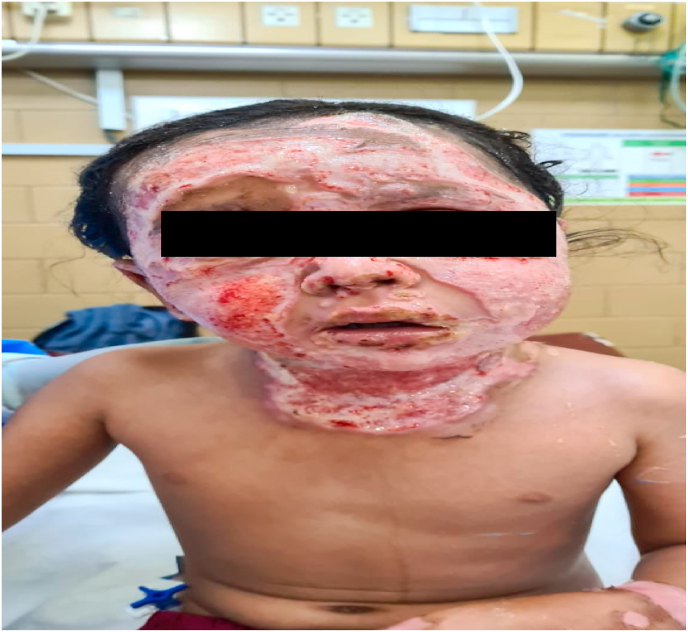
Case 2: 5-year-old boy with face and neck lesions.

A 5-year-old boy presented with 35% TBSA burns involving his head, neck, front of chest, and upper and lower limbs. His vitals on admission were HR 100 bpm, O2 saturation 98%, afebrile, and BP 100/70 without any respiratory distress. Initial chest x-ray (CXR) and blood gases were normal. A central venous line (CVP) and Foley catheter were passed and adequate dressing was applied. The patient was managed with fluid resuscitation as per the Parkland formula and was given injections of meropenem 330 mg IV Q8H, moxifloxacin 150 mg IV OD, esomeprazole 15 mg IV OD, tetanus toxoid, and paracetamol SOS. The patient was monitored closely in the ICU and daily dressing was performed with alternate day debridement. Until day 5, the patient did not develop any skin lesions. Further, his blood and tissue cultures on day 5 were negative. On day 6, he started developing a high-grade fever and punched-out skin lesions (Fig. 3). Cultures were sent immediately thereafter and with results positive for fungal growth, he was placed on amphotericin B.

### 1.3 Case 3

**Fig. 4:**
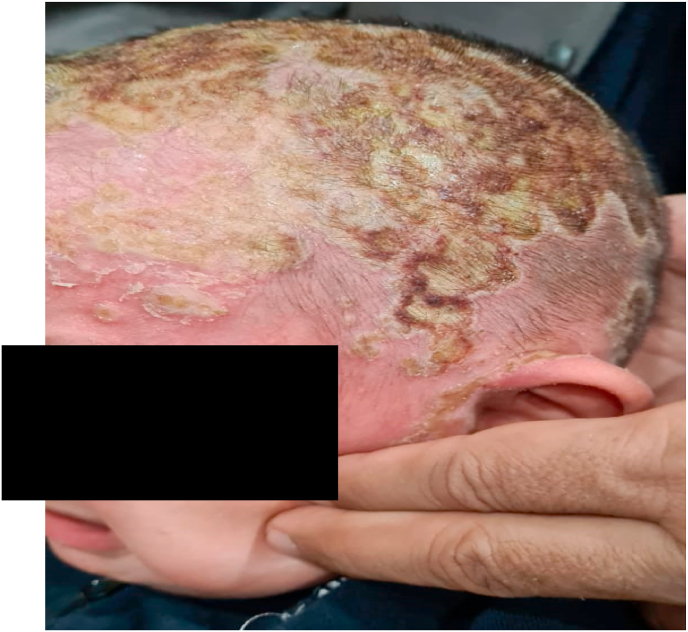
Case 3: 2.5-year-old boy with scalp burns.

A 2-and-a-half-year-old boy presented with 15% TBSA burns due to boiling water on his scalp, face, chest, and shoulders. Upon initial examination the boy was irritable but stable with a HR 120 bpm, normotensive, and afebrile without any respiratory distress. He was given a tetanus toxoid injection with injections of amoxiclav 300 mg IV Q8H, amikacin 150 mg IV Q12H, and the affected area was covered with polymyxin B (Polyfax) ointment. The patient was monitored in the burn unit. On day 7 of his admission, he developed a fever and skin lesions similar to Case 1 (Fig. 4) and fungal cultures were sent which returned positive, prompting voriconazole administration. He remained in the burn unit until he was discharged.

### 1.4 Case 4

An 11-year-old boy presented with 55% TBSA burns involving the head, neck, buttocks, anterior and posterior trunk. The patient had a HR 110 bpm, O2 saturation 92%, afebrile, normotensive, and blood gas readings indicating mild acidosis. A CVP line was passed and tracheostomy was performed due to the severity of inhalational injury. Cleaning and dressing of the wounds were performed. He was administered injections of meropenem 500 mg IV Q8H, moxifloxacin 200 mg IV OD, ticaclav 1.6 g IV Q8H, esomeprazole 20 mg IV OD, and paracetamol IV SOS. The patient was admitted to the ICU and monitored closely. Initial blood cultures were negative. Around day 5, however, the patient developed a high grade fever and punched-out eruptions on his burn areas after which fungal and tissue cultures were promptly sent out which showed *E. coli* and Fusarium growth. The patient's condition deteriorated within the next 2 days, and he developed severe acute respiratory distress syndrome (ARDS) confirmed on high resolution computed tomography (HRCT) and CXR. On day 7 the patient was intubated and put on a ventilator. He expired on day 9.

### 1.5 Case 5

**Fig. 5:**
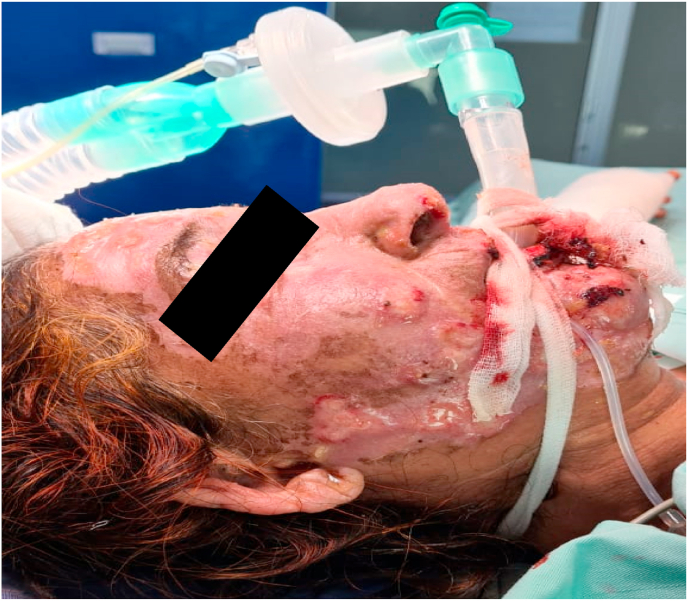
Case 5: 57-year-old female with 30% TBSA.

A 57-year-old female presented with 30% TBSA burns on the face and both upper and lower limbs. On examination the patient had a HR 92 bpm, O2 saturation 96%, BP 160/90, and was afebrile. She was administered IV fluids per the Parkland formula, injections of meropenem 1 g IV Q8H, moxifloxacin 400 mg IV OD, esomeprazole 40 mg IV OD, paracetamol SOS, tetanus toxoid, nebulization TDS, and oxygen. She was admitted to the ICU and debridement was performed every alternate day under general anesthesia (GA). On day 3, she developed skin lesions (Fig. 5); tissue and blood cultures were positive for Fusarium. She was subsequently put on amphotericin B and responded very well. She had skin grafting performed during her admission, which was a total of 4 weeks.

### 1.6 Case 6

A 3-year-old girl presented with 22% TBSA burns on her trunk, thigh, and perineum. On examination she was normotensive and afebrile with no respiratory distress. Her CXR was also normal. Initial treatment included injections of amoxiclav 300 mg IV Q8H, moxifloxacin 150 mg IV OD, esomeprazole 15 mg IV OD, paracetamol IV SOS, and IV fluids as per the Parkland formula. She was admitted to the ICU where alternate day debridement was done under GA. On day 2 she developed skin lesions that revealed positive growth for Fusarium dimerum on Fungal culture. She was put on amphotericin B, to which the patient responded, improved, and was subsequently discharged.

### 1.7 Case 7

**Fig. 6:**
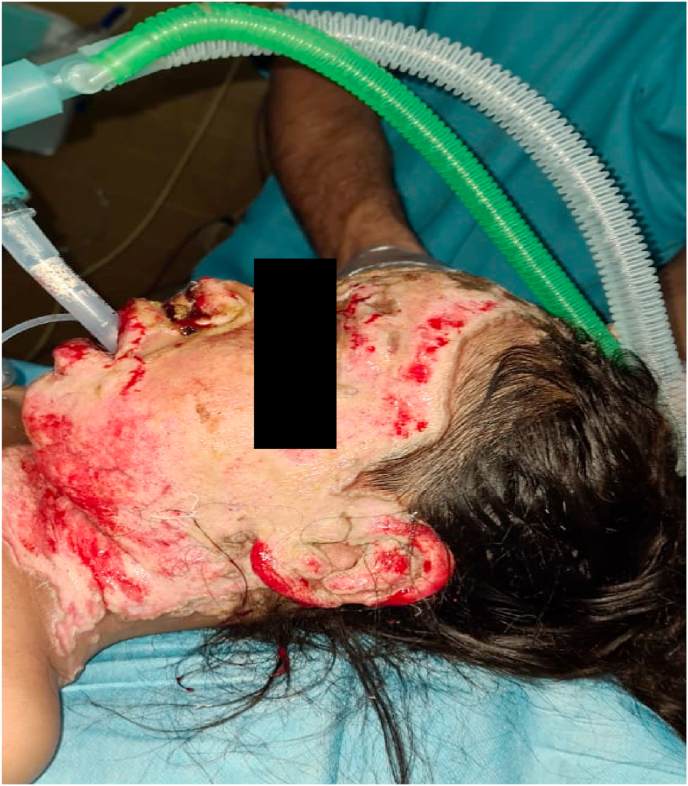
Case 7: A 5-year-old female child with 22% TBSA burns.

A 5-year-old girl presented with 30% TBSA burns involving the upper limbs, abdomen, chest, and lower limbs. On initial examination the girl was irritable but stable with a HR 110 bpm, normotensive, and afebrile without any respiratory distress. She was given injections of tetanus toxoid, amoxiclav 300 mg IV Q8H, and amikacin 150 mg IV Q12H. Polyfax ointment was applied to the affected areas. The patient was monitored in the burn unit. On day 7 she developed skin lesions (Fig. 6); fungal cultures returned positive and she was administered a regimen of voriconazole. She remained in the burn unit until she was discharged.

### 1.8 Case 8

The last Case is a 47-year-old male who presented with 10% TBSA deep dermal burns on both hands and forearms. Upon examination the patient had a HR 82 bpm, was normotensive and afebrile. He was administered injections of amoxiclav 1.2 g Q8H, esomeprazole 40 mg IV OD, moxifloxacin 400 mg IV OD, ketorolac tromethamine (Toradol) SOS, and IV fluids were administered according to the Parkland formula. Over the course of his stay in the burn unit he had debridement performed. On day 3 of admission, the patient exhibited skin lesions positive for Fusarium growth on fungal culture and histopathological sample analysis. He was thus treated with voriconazole. After the lesions resolved he received a skin graft and was subsequently discharged.

## 2 Discussion

F. dimerum is a very rare opportunistic fungus, much like other Fusarium species, that causes infections in immunocompromised patients such as the burn victims in our Case series. Considering its rapid spread across the admitted patients, its infectivity is alarming. One recent study regarding the variability of pathogens commonly encountered in burn lesions revealed that 39% of wounds were infected by mold or Candida [11]. Analysis of the mold growths revealed the Fusarium species as the most common occupant, second only to Aspergillus.

Infection of burns caused by Fusarium has been previously observed in Case studies. The most recent report came from Vietnam, where a 24-year-old patient with 25% TBSA underwent treatment with intravenous fluids, parenteral antibiotics and antifungal drugs, and topical silver sulfadiazine ointment alongside surgical debridement and skin transplantation [5]. His swab cultures turned out positive for multiple organisms and appropriate treatment was thus administered. Unfortunately, the septicemia caused by *P. aeruginosa* led to multiorgan failure and subsequent death. Follow up cultures produced white colonies with many wide crescent-shaped macroconidia, characteristic of Fusarium on microscopic examination.

In 2019, a similar Case report was published, where a 55-year-old male had sustained a 35% TBSA full-thickness burn to his back, upper chest, and bilateral upper extremities [4]. Following the initial intubation, resuscitation, and treatment with hyperbaric oxygen,. escharotomies were performed: H. influenzae and *S. aureus* were isolated from sputum and blood cultures respectively. He was treated with piperacillin/tazobactam and vancomycin along with excision of burn wounds. On day 12 post-admission, he developed septic shock and acute kidney injury requiring hemodialysis. Tissue cultures identified a mold: F. solani. Despite initiation of aggressive operative debridement and antifungal therapy, the patient's condition worsened, ultimately requiring amputation below the left elbow followed by split-thickness skin grafting. After the surgery, the patient's status improved remarkably.

A recent review based on the Case reports of 44 burn patients revealed that the mortality rate for fungal infection was high (27.27%), with the majority of fatalities resulting from non-Candida species including Fusarium [12]. This recapitulates the findings of a previous retrospective study of 168 patients, which described a 24% mortality among patients infected with filamentous fungi; of note, Fusarium was responsible for 50% of deaths [13]. This in and of itself highlights the need to actively consider fungal infection when monitoring burn injuries. Effective and early treatment protocols directed towards eradicating the fungi can lead to a substantial decrease in mortality that are associated with dissemination.

High-dose intravenous amphotericin B is considered the mainstay for the treatment of fusariosis [14]. In patients intolerant to or have conditions that contraindicate the use of amphotericin B, voriconazole has been shown to be an effective alternative, as its in vitro and in vivo activity against Fusarium has been proven [15]. Posaconazole is yet another drug that has been suggested as a therapy for fusariosis. In fact, the United States Food and Drug Administration (USFDA) has approved voriconazole and posaconazole as standard therapy for fusariosis in immunocompromised individuals. Posaconazole, for example, is dosed at 200 mg three times a day per os [16]. Other -azole antifungal drugs such as itraconazole, albaconazole, and ravuconazole are thought to be of limited use for the treatment of Fusarium spp [17]. In this Case series, the 7-day regimen of amphotericin B and voriconazole was effective for the recovery of all but one patient, who did not survive due to extensive burns and septicemia.

Pertinently, all the cases have been reported in line with the PROCESS guidelines [18].

## 3 Conclusion

Our Case series focuses on the *Fusarium* spp., particularly *F. dimerum,* a species of opportunistic fungal pathogens in burn patients. Prompt treatment plans, appropriate wound care, and empirical antibiotic regimens for burn ward admissions have considerably lowered mortality rates for this patient population. Currently, there is a keen interest in the identification of new isolates from the *Fusarium* spp. and their antifungal susceptibility patterns, as this will undoubtedly fill in the gaps regarding the complications of opportunistic fungal infections. The high risk of dissemination necessitates prompt screening using histologic and mycologic diagnostics.

Fusariosis is a rare fungal infection and thus does not have substantial clinical evidence regarding its infectivity, management, and treatment options. Our study aims to improve the knowledge concerning the epidemiology of the Fusariosis and to emphasize the importance of keeping a high clinical suspicion in burn patients.

## Ethical approval

NA.

## Sources of funding

NA.

## Author contribution

SNK, NR, ZAK: conceived the idea, designed the study, and drafted the manuscript, AN, AMK, TA, AG: conducted literature search and created the illustrations, TK, VRN, AA, RA: revised the manuscript critically and gave the final approval.

## Registration of research studies

Name of the registry: NA.

Unique Identifying number or registration ID: NA.

Hyperlink to your specific registration (must be publicly accessible and will be checked): NA.

## Guarantor

Talal Almas.

## Provenance and peer-review

Not commissioned, externally peer-reviewed.

## Consent

Obtained.

## Disclosures

NA.

## Declaration of competing interest

NA.
